# Optimization of postoperative surveillance protocols in upper tract urothelial cancer: A retrospective cohort study

**DOI:** 10.3389/fonc.2023.1143030

**Published:** 2023-03-14

**Authors:** Andrea Katharina Lindner, Martin Pichler, Sarah Maier, Hanno Ulmer, Thomas Gorreri, Anna Katharina Luger, Dominik A. Barth, Andreas Seeber, Florian Kocher, Renate Pichler

**Affiliations:** ^1^ Department of Urology, Comprehensive Cancer Center Innsbruck, Medical University of Innsbruck, Innsbruck, Austria; ^2^ Division of Oncology, Department of Internal Medicine, Medical University of Graz, Graz, Austria; ^3^ Translational Oncology, University Hospital of Augsburg, Augsburg, Germany; ^4^ Institute of Medical Statistics and Informatics, Medical University of Innsbruck, Innsbruck, Austria; ^5^ Department of Radiology, Medical University of Innsbruck, Innsbruck, Austria; ^6^ Department of Hematology and Oncology, Comprehensive Cancer Center Innsbruck, Medical University of Innsbruck, Innsbruck, Austria

**Keywords:** surveillance, upper tract carcinoma, follow up, recurrence, kidney sparing surgery

## Abstract

Upper tract urothelial carcinoma (UTUC) is an aggressive disease that is managed by radical or organ-sparing surgery. High recurrence rates require early detection and strict follow-up (FU) protocols. Recommendations are assigned to a low level of evidence. Our aim was to identify time-to-tumor recurrence, analyze the temporal relation to recommended FU regimens, and provide a critical proposal for further surveillance. This retrospective study included 54 patients receiving radical nephroureterectomy (RNU) in high-risk UTUC and 14 patients assigned to kidney-sparing surgery (KSS) with low-risk disease. FU surveillance protocols consisted of close intervals irrespective of the received type of surgery. In total, 68 patients were included with a median FU of 23 months. Mean overall survival (OS) was significantly shorter in RNU compared to KSS (*P* = .027). Recurrence in the bladder and/or upper urinary tract (UUT) was 57.1% in KSS and 38.9% after RNU (*P* = .241). Mean recurrence-free survival (RFS) was significantly shorter in RNU patients compared to KSS (22.4 *vs.* 47.9 months, *P = .*013), and 76.2% of the recurrences in the RNU group occurred in the first postoperative year. UUT recurrence was diagnosed after a median of 3.0 (RNU) and 25.0 (KSS) months. There was a frequent onset of metastases in the RNU group, with 85.7% in the first year compared to the KSS group with 50%. Multivariable regression analysis showed that the tumor stage was the parameter independently related to OS (*P = .*002), RFS (*P = .*008), and metastasis-free survival (MFS, *P* = .002). In conclusion, surveillance of UTUC should be adapted to real-time occurrence patterns. Strict imaging protocols are recommended in the first two years irrespective of the method of surgery. As recurrence is equally distributed over the years after KSS, cystoscopy should be offered regularly for five years and diagnostic URS for three years. After RNU, cystoscopies should be decreased to yearly intervals after year three. Contralateral UUT should also be examined after RNU.

## Introduction

Between 5 and 10% of all urothelial tumors occur in the renal pelvis and ureter, considering that upper urothelial tract carcinoma (UTUC) is a rare urological disease (1). Up to 60% of UTUCs present with invasive growth at primary diagnosis, with high tumor stage presentation and high cancer-specific mortality. Urothelial carcinoma is the most common histopathological finding in UTUC, with divergent squamous differentiation described in up to 15% of cases. Variant histologies, particularly of the sarcomatoid subtype, present with high-grade components and a worse survival when compared with pure urothelial cancer (1). High recurrence risk rates, especially in the lower urinary tract (2–4), give UTUC the characteristics of a highly destructive disease (5, 6). The absence of the muscularis propria layer in the upper urinary tract (UUT) leads to poor prerequisites of tumor staging in primary bioptic diagnostics and is causal for early spreading growth.

An overall 5-year survival of 57% underlines the imperative importance of the implementation of an efficient follow-up (FU) scheme to detect recurrences as early as possible with a primary curative intention and yet not exposing the patient to unnecessary examination risks. The upmost goal is to obtain a curative setting after diagnosis and primary surgery for UTUC, which can be achieved though accurate and close FU. The impact of standardized regular surveillance on the better oncological prognosis has already been investigated in several malignancies, including primary urothelial cancer of the bladder (7, 8). Strict FU protocols detecting asymptomatic recurrence, occurring in up to 57% after RNU, have been shown to potentially improve survival rates compared to patients with symptomatic recurrence (9). This again points out the objective for regular surveillance in UTUC to achieve a better outcome based on early recurrence detection. To achieve this, and due to the rarity and heterogeneous occurrence of the UTUC, the implementation of prospective randomized studies remains unfeasible, thus giving retrospective studies with standardized strict follow-up protocols a key imperative role.

Open or laparoscopic radical nephroureterectomy (RNU) with a bladder cuff was the gold-standard therapy for all cases of UTUC for a long time. In low-risk stratified tumors at primary biopsy, kidney-sparing surgery (KSS) has been shown to hold similar survival rates compared to RNU and is now the primary treatment option in low-risk settings and in selected cases with high-risk patients (1, 10). Although KSS brings the advantage of organ preservation, it involves frequent invasive ipsilateral FU at intervals that have been difficult to clearly define to date. Recommendations for FU care in the international guidelines are still assigned to a low level of evidence only, mainly due to the small retrospective cohort sizes (1, 6). Due to the small number of affected patients and a consistently inhomogeneous pattern of institutional FU regimens, few studies on aftercare intervals are available. The known 22 - 47% high risk of bladder recurrence demands meticulous and stringent monitoring of the lower urinary tract in particular, for recurrence is a key component of further oncological outcome (11). Cystoscopy remains the gold standard for the screening and detection of bladder cancer, and newer technologies such as photodynamic diagnostics have already been implemented to diagnose invisible lesions (12).

Postoperative surveillance of UTUC includes cystoscopy and cytology (13), as well as imaging consisting of abdominal and chest computed tomography (CT) (1, 14). KSS additionally requires regular ureterorenoscopy (URS), which always requires general anesthesia, thus underlying the importance of excellent patient compliance (15). A recent metaanalysis has evaluated the intensity and duration of oncologic FU after RNU in high-risk patients with and without a previous finding of urothelial cancer in the bladder. The group proposes to extend semi-annual imaging and tailor cystoscopies in this patient group to a yearly basis after the fourth year of FU, with which they estimate an adaptation of the current guidelines (16).

The aim of the current study was to identify the time patterns of recurrence in the bladder and UUT, analyze their temporal relation to recommended FU regimens, and provide a critical proposal for the further surveillance of patients with UTUC.

## Methods

This is a retrospective observational study based on an Austrian urooncology cancer database. Analysis was performed for the period between January 2010 and January 2020. The consent of the local ethics commission of the Medical University Innsbruck was obtained with the approval number 1053/2021.

### Patient population and pathological staging

Medical records of patients diagnosed with primary non-metastatic UTUC followed by either radical or kidney-sparing surgery at our department between January 2010 and January 2020 were reviewed retrospectively. All patients with a confirmed histopathology of primary urothelial cancer of the upper urothelial tract without a history of previous bladder cancer and eligible FU data of at least two years at our outpatient department were included. Patients with no definitive histopathology, simultaneous urothelial cancer of the bladder, those who were followed-up elsewhere postoperatively, or who had evidence of distant or local metastasis on imaging before surgery were excluded. Tumor staging was performed according to the 2016 tumor–node–metastasis (TNM) classification and grading, referring to the World Health Organization and International Society of Urological Pathology categorization (1).

### Primary tumor treatment

In a preoperative evaluation, patients were assigned to a low- or high-risk cohort. A definition of ‘high-risk’ UTUC was applied according to the current guidelines of the European Association of Urology (EAU), including following criteria: multifocal disease, tumor size > 2 cm, high-grade cytology or biopsy, local invasion on imaging, variant histology, presence of hydronephrosis, or previous radical cystectomy (1). Patients with high-risk disease received primary radical surgery by means of open or laparoscopic radical nephroureterectomy (RNU) with a bladder cuff. Kidney-sparing surgery (KSS) was performed in patients with ‘low-risk’ tumors. To avoid distorting results between low- and high-risk, we excluded all patients with isolated incidents such as single kidneys, bilateral tumor findings, or diverging treatment decisions. KSS was defined as endoscopic ablative management or a ureterectomy with an intraoperative frozen section and ureteral reimplantation if located in the distal ureter (10). Staging was conducted preoperatively by means of a thoracoabdominal computer tomography (CT) scan. Lymph node dissection (LND) was performed at the discretion of the surgeon, with a template-based extensive completion whenever possible in high-risk settings (17). Concerning resection margin status, R0 corresponds to complete resection without a traceable tumor rest, R1 to microscopic tumor residual, and R2 to macroscopic tumor residual. Neoadjuvant chemotherapy was not administered, and no included patient received intravesical mitomycin C, as the evidence-based use of recommended postoperative intravesical chemotherapy instillation took a lift in 2015 and was only afterwards steadily implemented in our institutional practice (18).

### Follow-up and recurrence

**Figure 1:**
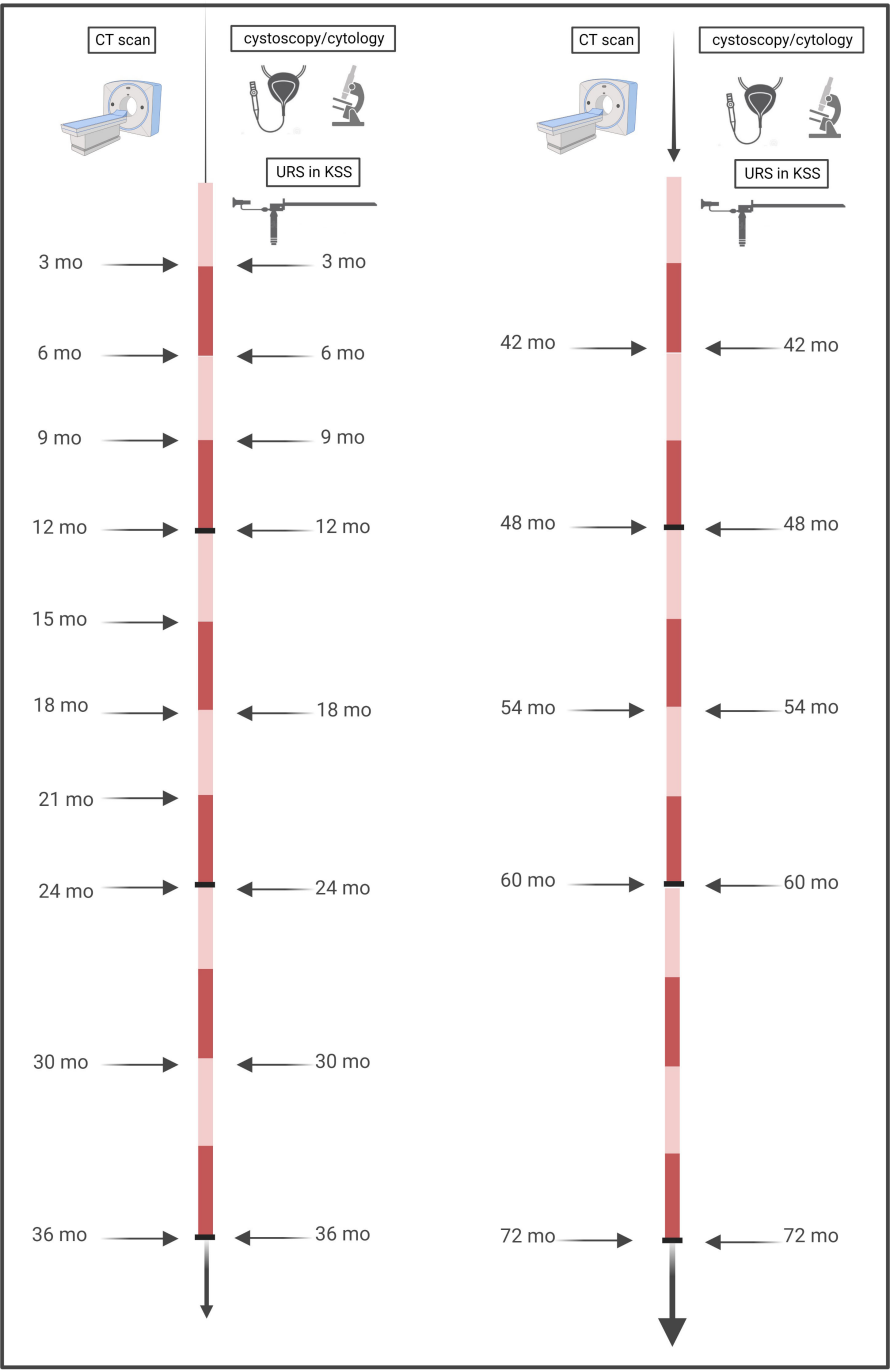
Timeline of performed follow-up. Our institutional practice for all (low-risk and high-risk) patients, irrespective of the method of surgery, consisted of close-meshed imaging intervals of three months for the first two years, with six-monthly continuation thereafter until the fifth year, and then yearly. Cystoscopies with ipsilateral URS for KSS were performed every three months in the first year, followed by six-monthly intervals until the fifth year, and then yearly.

Tumor recurrence was defined as reappearance of urothelial cancer in the bladder or upper urinary tract (UUT), which was confirmed histologically either by transurethral resection of the bladder or biopsy during ureterorenoscopy. According to our institutional practice and due to known higher aggressiveness in high-risk and common recurrence, all patients received the same FU intervals. These consisted of physical examination and thoracoabdominal CT with a contrast medium and urographic phase at three months during the first year, every six months between the second and fifth year, and yearly thereafter. Cystoscopy and urinary cytology including ipsilateral URS after KSS were performed every three months in the first two years, then every six months up to year five of follow-up, and then yearly, as seen in **Figure 1**.

### Statistical analysis

Time-to-event endpoints, including overall survival, cancer-specific survival, recurrence-free survival, and metastasis-free survival, were analyzed using life tables, Kaplan–Meier with log-rank testing, and Cox proportional hazards regression analyses. Two sets of Cox models were fitted: firstly age- and sex-adjusted models and secondly multivariably adjusted models with the operation type, pT-stadium, R-status, and tumor grade as potential predictors. The variable selection for the multivariable model was based on statistical significance (*P* <.05) in the age- and sex-adjusted model. Hazard ratios and their 95% confidence intervals were reported. In addition, chi-square or Fisher’s exact test for categorical variables and T testing for continuous variables were performed. All other analyses were conducted in a descriptive way. P-values < 0.05 were considered to indicate statistical significance. IBM SPSS Statistics 26 (IBM Corp, Version 28.0, released 2021, Armonk, NY) was used for statistical calculations. Figures were created with IBM SPSS Statistics 26 and BioRender (Toronto, ON).

## Results

### Patient and tumor characteristics

A total of 68 patients were diagnosed and treated with primary non-metastatic UTUC at our institution. The mean (± SD) age was 69 (± 9) years; 58.8% (n=40) were male and the median (range) FU was 23.0 (0-119) months in the overall cohort. A total of 20.6% (n=14) underwent KSS, and 79.4% (n=54) received RNU as the primary surgical treatment. Overall, UTUC of the renal pelvis was found in 56.7% of patients; 43.3% were detected in the ureter. The histopathological results revealed urothelial carcinoma in all patients, with sarcomatoid differentiation in two patients. All patients (n=14) receiving KSS had UTUC that was found and limited to the ureter. In patients receiving RNU, the renal pelvis was the most common tumor localization in 71.7% (n=38), and the remaining 28.3% had UTUC found in the ureter (*P <.001*). A total of 18 (75%), 5 (20.8%), and 1 (4.2%) of patients were staged as pTa, pT1, and pT2 in the primary biopsy during diagnostic URS, respectively. Only in patients receiving RNU were high-grade tumors present at biopsy in 37% of the cases. All cases of KSS had a low-grade tumor stage in the primary biopsy. Of all patients, 3 (4.4%), 24 (35.3%), 9 (13.3%), 7 (10.3%), and 25 (36.8%) presented with the final tumor staged as pTis, pTa, pT1, pT2, and pT3/4, respectively. Final tumor grading after surgery resulted in 57.1% (n=8) and 61.1% (n=33) high-grade tumors, with the mainly classified grade G1/2 in 78.6% (n=11) and 79.6% (n=43) in KSS and RNU, respectively, representing the low diagnostic value of the primary biopsy of the UUT. Tumor-free resection margins (R0) were achieved in 92.9% of patients receiving KSS and 83.3% in RNU. Lymphadenectomy (LAD) revealed negative findings (pN0) in all evaluated patients treated with KSS and positive findings in 44.4% after receiving RNU (*P = .031)*. **Table 1** summarizes the descriptive and histopathological patient characteristics according to the surgical approach-related differences.

**Table 1:** Descriptive patient, histopathological, and treatment characteristics of the study population.

Characteristics	Overall (N=68)	KSS (N=14)	RNU (N=54)	p-value*
**Age [years], mean (SD)**	69.38 (± 9.09)	71.50 (± 8.30)	68,83 (± 9.27)	0.332
**Female sex, n (%)**	28 (41.2%)	4 (28.6%)	24 (44.4)	0.368
**Tumor stage at biopsy, n (%)**				1.000
** pTa**	18 (75%)	5 (83.3%)	13 (72.2%)	
** pT1**	5 (20.8%)	1 (16.7%)	4 (22.2%	
** pT2**	1 (4.2%)	–	1 (5.6%)	
Tumor grade at biopsy, n (%)*				**0.016**
** Low-grade**	30 (75%)	13 (100%)	17 (63%)	
** High-grade**	10 (25%)	–	10 (37%)	
**Localization, n (%)**				**< 0.001**
** Renal pelvis**	38 (56.7%)	–	38 (71.7%)	
** Ureter**	29 (43.3%)	14 (100%)	15 (28.3%)	
**Final Tumor stage (pT), n (%)**				0.095
** pTIS**	3 (4.4%)	2 (14.3%)	1 (1.9%)	
** pTa**	24 (35.3%)	6 (42.9%)	18 (33.3%)	
** pT1**	9 (13.3%)	2 (14.2%)	7 (13%)	
** pT2**	7 (10.3%)	2 (14.3%)	5 (9.3%)	
** pT3/4**	25 (36.8%)	2 (14.3%)	23 (42.6%)	
**Tumor grade, n (%)**				1.000
** Low-grade**	27 (39.7%)	6 (42.9%)	21 (38.9%)	
** High-grade**	41 (60.3%)	8 (57.1%)	33 (61.1%)	
Tumor grade, n (%)*				1.000
** G1/2**	8 (36.4%)	2 (40%)	6 (35.3%)	
** G3/4**	14 (63.6%)	3 (60%)	11 (64.7%)	
**R status, n (%)**				0.739
** R0**	58 (85.3%)	13 (92.9%)	45 (83.3%)	
** R1**	9 (13.2%)	1 (7.1%)	8 (14.8%	
** R2**	1 (1.5%)	–	1 (1.9%)	
**Lymphadenectomy, n (%)**				0.129
** No**	42 (61.8%)	6 (42.9%)	36 (66.7%)	
** Yes**	26 (38.2%)	8 (57.1%)	18 (33.3%)	
**pN status, n (%)**				**0.031**
** Negative**	18 (69.2%)	8 (100%)	10 (55.6%)	
** Positive**	8 (30.8%)	–	8 (44.4%)	

*Findings evaluated for patients with pathological results present. Bold values mean: p < 0.05.

### Survival outcomes

**Figure 2:**
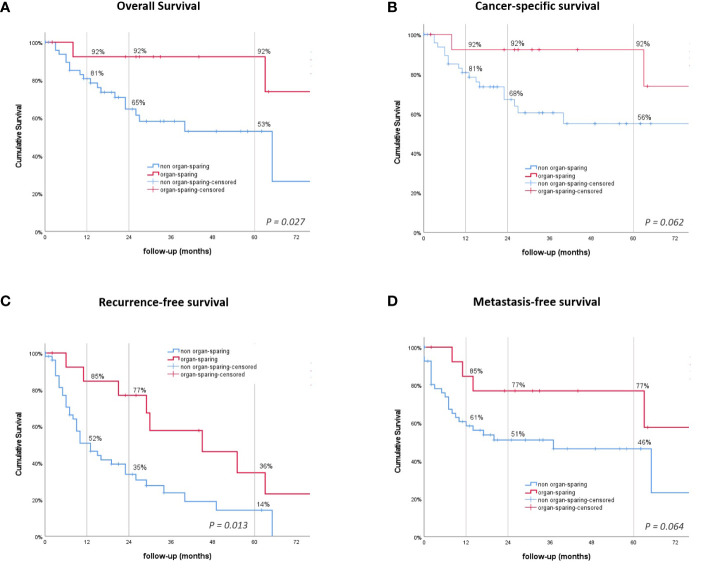
Kaplan–Meier survival curves. **(A)** Overall survival (OS). **(B)** Cancer-specific survival. **(C)** Recurrence-free survival (RFS). **(D)** Metastasis-free survival. *P-*values by log-rank.

In patients receiving RNU, the mean overall survival (OS] was significantly shorter, at 55.7 months compared to 100.1 months after KSS (*P = .027*, **Figure 2A** *).* Cancer-specific survival (CSS) was trending, but not statistically significant (*P = .062*), with a shorter mean time of 70.9 compared to 100.1 months for the RNU and KSS groups (**Figure 2B**). The 1-, 2-, and 5-year OS and CSS rates after RNU were 81%, 65%, and 53%, and 81%, 68%, and 56%, respectively. The OS and CSS rates for KSS were 92% unchanged across the three time points (**Table 2**).

**Table 2:** Cumulative 1 -, 2 -, and 5 - year survival.

		1-year survival	2-year survival	5-year survival
***OS***	**overall**	83%	72%	63%
	**RNU**	81%	65%	53%
	**KSS**	92%	92%	92%
***CSS***	**overall**	83%	74%	65%
	**RNU**	81%	68%	56%
	**KSS**	92%	92%	92%
***RFS***	**overall**	59%	44%	19%
	**RNU**	52%	35%	14%
	**KSS**	85%	77%	36%
***MFS***	**overall**	66%	57%	53%
	**RNU**	61%	51%	46%
	**KSS**	85%	77%	77%

In the univariate sex- and age-adjusted analysis, the pathological tumor stage was associated with OS (hazard ratio [HR] = 2.16, 95% CI: 1.43-3.27, *P <.001*), CSS (HR = 2.40, 95% CI: 1.49-3.86, *P <.001*), RFS (HR = 1.29, 95% CI: 1.09-1.53, *P = .003*), and metastasis-free survival (MFS) (HR = 1.87, 95% CI: 1.43-2.46, *P <.001*). The tumor grade (HR = 4.43, 95% CI: 1.28-15.36, *P = .019*) was a predictor of CSS, whereas the type of surgery (HR = 2.64, 95% CI: 1.19-5.85, *P = .016*) influenced RFS. MFS was additionally associated with R status (HR = 3.35, 95% CI: 1.41-7.98, *P = .006*) and tumor grading (HR = 5.96, 95% CI: 2.02-17.02*, P = .001*). Moreover, the operation type (HR = 4.62, 95% CI: 1.04-20.52, *P = .044*) and tumor grading (HR = 3.41, 95%CI: 1.13-10.26, *P = .029*) influenced OS significantly (**Table 3**).

**Table 3:** Age- and sex-adjusted predictors for patient survival; HRs estimated with Cox proportional hazards regression analysis.

	Overall survival			Cancer-specific survival			Recurrence-free survival			Metastasis free survival		
Predictors	HR	95% CI	p-value	HR	95% CI	p-value	HR	95% CI	p-value	HR	95% CI	p-value
Operation type (RNU *vs*. KSS)	4.621	01.040-20.525	**0.044**	3.778	0.841-16.974	0.083	2.644	1.195-5.849	**0.016**	2.596	0.888-7.587	0.081
pT-stage (per unit)	2.163	1.432-3.268	**< 0.001**	2.401	1.492-3.865	**< 0.001**	1.292	1.093-1.526	**0.003**	1.873	1.424-2.464	**< 0.001**
R-Status (R1/2 *vs*. R0)	2.103	0.751-5.888	0.157	1.918	0.621-5.923	0.258	1.772	0.809-3.882	0.152	3.354	1.409-7.984	**0.006**
Tumor grade (high *vs*. low)	3.406	1.131-10.259	**0.029**	4.433	1.279-15.364	**0.019**	1.667	0.873-3.182	0.121	5.960	2.018-17.015	**0.001**

Bold values mean: p < 0.05.

In the multivariate analysis, the tumor stage was the sole parameter, independently related to RFS (HR = 1.26, 95% CI: 1.06-1.49, *P = .008*), CSS (HR = 2.37, 95% CI: 1.42-3.97, *P = .001*), MFS (HR = 1.63, 95% CI: 1.19-2.21, *P = .002*), and OS (HR = 2.07, 95% CI: 1.30-3.28*, P = .002*). Moreover, the method of surgery independently influenced RFS (HR = 2.353, 95% CI: 1.037-5.340, *P = .041*) (**Table 4**).

**Table 4:** Multivariably adjusted predictors for patient survival; HRs estimated with Cox proportional hazards regression analysis.

	Overall survival			Cancer-specific survival			Recurrence-free survival			Metastasis free survival		
Predictors	HR	95% CI	p-value	HR	95% CI	p-value	HR	95% CI	p-value	HR	95% CI	p-value
Operation type (RNU *vs*. KSS)	2.369	0.502-11.184	0.276				2.353	1.037-5.340	**0.041**			
pT-stage (per unit)	2.065	1.301-3.276	**0.002**	2.369	1.416-3.965	**0.001**	1.256	1.061-1.486	**0.008**	1.625	1.197-2.206	**0.002**
R-Status (R1/R2 *vs*. R0)										1.475	0.598-3.642	0.399
Tumor grade (high *vs*. low)	0.922	0.267-3.182	0.898	1.095	0.284-4.228	0.895				2.395	0.732-7.840	0.149

Bold values mean: p < 0.05.

### Recurrence patterns

**Figure 3:**
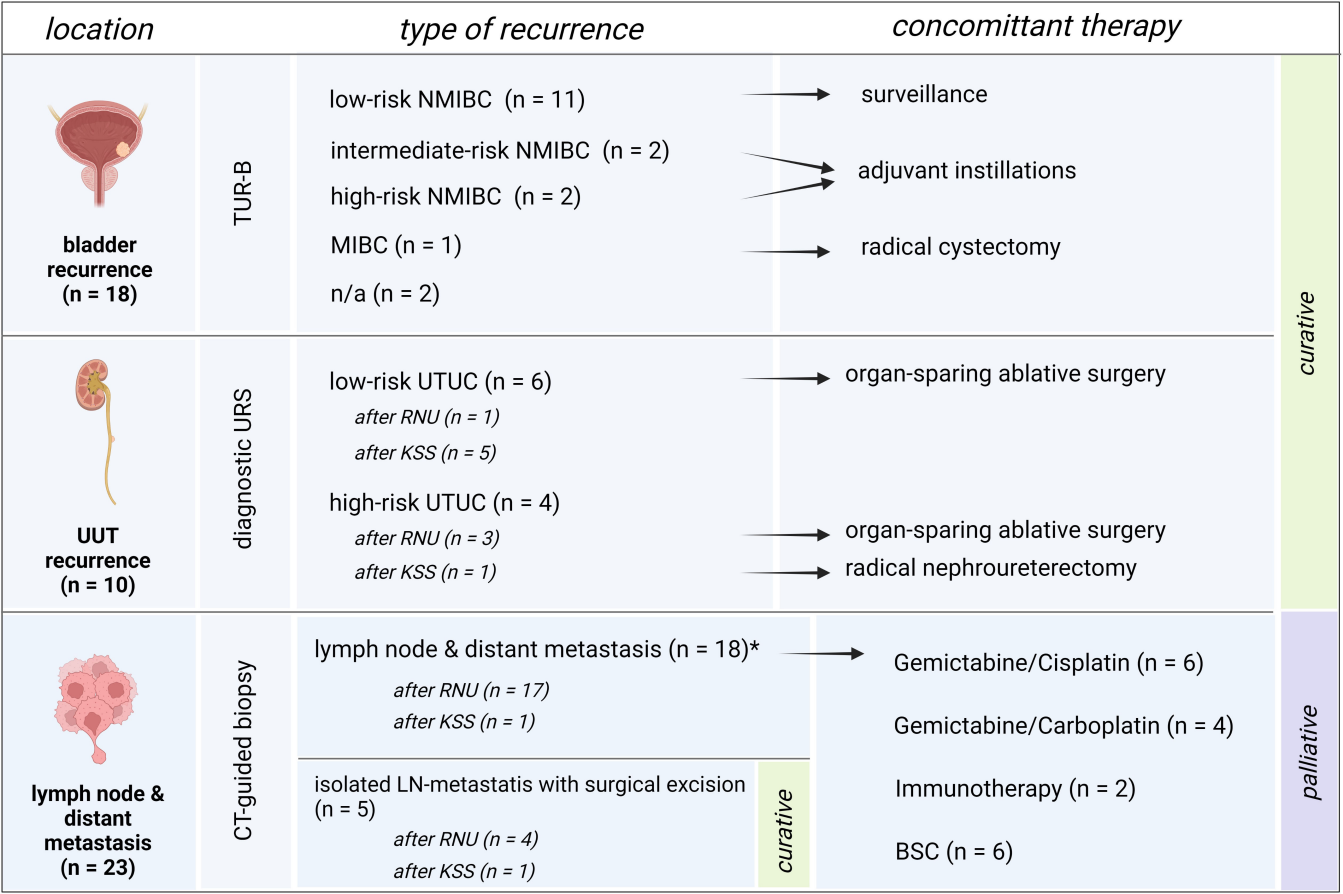
Overview of recurrences and subsequent therapies. Patient setting with bladder recurrence, recurrence of the upper urinary tract or distant metastasis, and their subsequent therapies. Bladder recurrence was treated in line with the treatment algorithm for primary bladder cancer. Low-risk UTUC recurrence of the upper urinary tract was treated with organ-sparing ablative surgery whenever possible. High-risk recurrence of the remaining UUT after RNU was treated with KSS whenever possible, and in KSS, radical surgery by means of RNU was applied whenever high-risk recurrence of the UUT occurred. Patients with solely lymph node recurrence were excised surgically with no further need of systemic therapy and therefore remained in a curative setting. Patients with metastatic disease were assigned to systemic therapy or best-supportive care depending on the patient status and therapy wishes.

The overall rate of recurrence in patients receiving KSS was 57.1% compared to only 38.9% after RNU (*P = .241*). The all-over time to recurrence showed a significant difference between the groups, with a median time to recurrence of 13 months after RNU and 45 months after undergoing KSS (*P = .013*, **Figure 2C**). The 1-, 2-, and 5-year RFS rates were 52%, 35%, and 14% for RNU and 85%, 77%, and 36% for KSS, respectively (**Table 2**). In detail, bladder recurrence was present in 28.1% (n=18) of patients, of which 16 underwent RNU and 2 underwent KSS after a median time of 9.0 (range 4-49) and 42.5 months (range 30-55), respectively. UUT recurrence was diagnosed in 10 patients: 4 patients after a median time of 3.0 months (range 0-9) after receiving RNU (contralateral) and 6 patients after a median time of 25.0 months (range 6-89) after organ-sparing surgery (ipsilateral). An overview of patients who had recurrence with their subsequent received treatment is shown *in* **Figure 3**.

### Lymph node and distant metastasis

Lymph node (LN) metastasis and/or distant metastasis occurred in 38.9% (n=21) of patients after radical surgery, while only 14.3% (n=2) were affected after organ preservation. The mean time to appearance of LN or distant metastasis was shorter, even if just not statistically significant, in the RNU group, with 46.6 months compared to 62.5 months in the KSS group *(P = .064*, **Figure 2D**). The 1 -, 2 -, and 5-year LN and MFS were shown to be 61%, 51%, and 46% after RNU and 85%, 77%, and 77% for the KSS cohort.

## Discussion

We evaluated patients diagnosed with primary non-metastatic UTUC without previous or synchronic bladder cancer after receiving radical or kidney-sparing surgery and close-meshed institutional FU in order to correlate their rates of recurrence with current guideline recommendations. The rarity of the cancer only gives short-to-mid-term survival outcomes from population-based studies (19), and the diagnostic and prognostic biomarkers still remain investigational (20, 21). Even strict FU regimens have shown results in up to 10 - 30% of instances of disease recurrence, including bladder recurrence, which are detected in some patients in advanced stages (22), thus making effective FU of uttermost importance. Previous studies have already shown that the primary risk factor for UTUC-related death is dependent on pathological stages (13, 23), as also seen in our population. It is known that UCC carries the highest treatment cost of all cancers (24–26), which underlines the importance that follow-up should be as efficient as possible, as overuse has been described in the past (26). Early detection of asymptomatic recurrences has already been shown to potentially improve survival rates compared to patients with symptomatic recurrence (9), which makes strict symptom-independent aftercare even more important. The current choice of duration of surveillance and invasiveness remains difficult, as there is no accurate statistical model taking the patient’s risk for recurrence characteristics over time into account and how the mode of surgery ultimately affects overall survival risk.

To make the analysis clear and comprehensive, we explicitly included high-risk patients who had undergone RNU and low-risk UTUC patients who had undergone KSS, excluding exceptional situations such as single kidneys or KSS in distal high-risk UTUC. Our data illustrate the difficulty of validating tumor staging and grading in the primary biopsy. In the KSS group, all patients were initially classified as low grade, but they revealed up to 57.1% of high-grade tumors in the final tumor grading. The rate of high-grade tumors at biopsy in RNU was much higher at 61.1%, with more frequent higher pT3/4 stages (64.7%) and more common positive lymph nodes in 44.4% of cases. In the comparison of the Kaplan–Meier-estimated survival curves, the KSS group was associated with better OS and CSS but demonstrated higher rates of recurrence compared to RNU. However, if bladder recurrence occurred, it was diagnosed significantly earlier in the RNU group, in line with previous findings in the literature (27). Literature on the evidence for optimal FU intervals is sparse, and given the rarity of UTUC, there is a paucity of guidelines on efficient surveillance. Present literature on FU is in part obtained from cohorts with synchronous bladder carcinomas, which biases the recurrence rate for patients with subsequently treated UTUC (28). We solely included patients without prior present bladder cancer to counteract this possible outcome bias. Additionally, most studies focus on follow-up strategies after RNU solely (16), lacking proposals for intervals in KSS. In our high-risk RNU cohort, the bladder recurrence rate was 38.9%, with a 76.2% chance of occurring in the first year and 9.5% in years two to three on FU. A cystoscopy should, therefore, be performed strictly every 3 months in the first two years, then every six months in the third year, and reduced to solely every year, as the incidence thereafter is in the very low percentage range, and cystoscopy remains an invasive procedure carrying the risk of infection (29).

**Figure 4:**
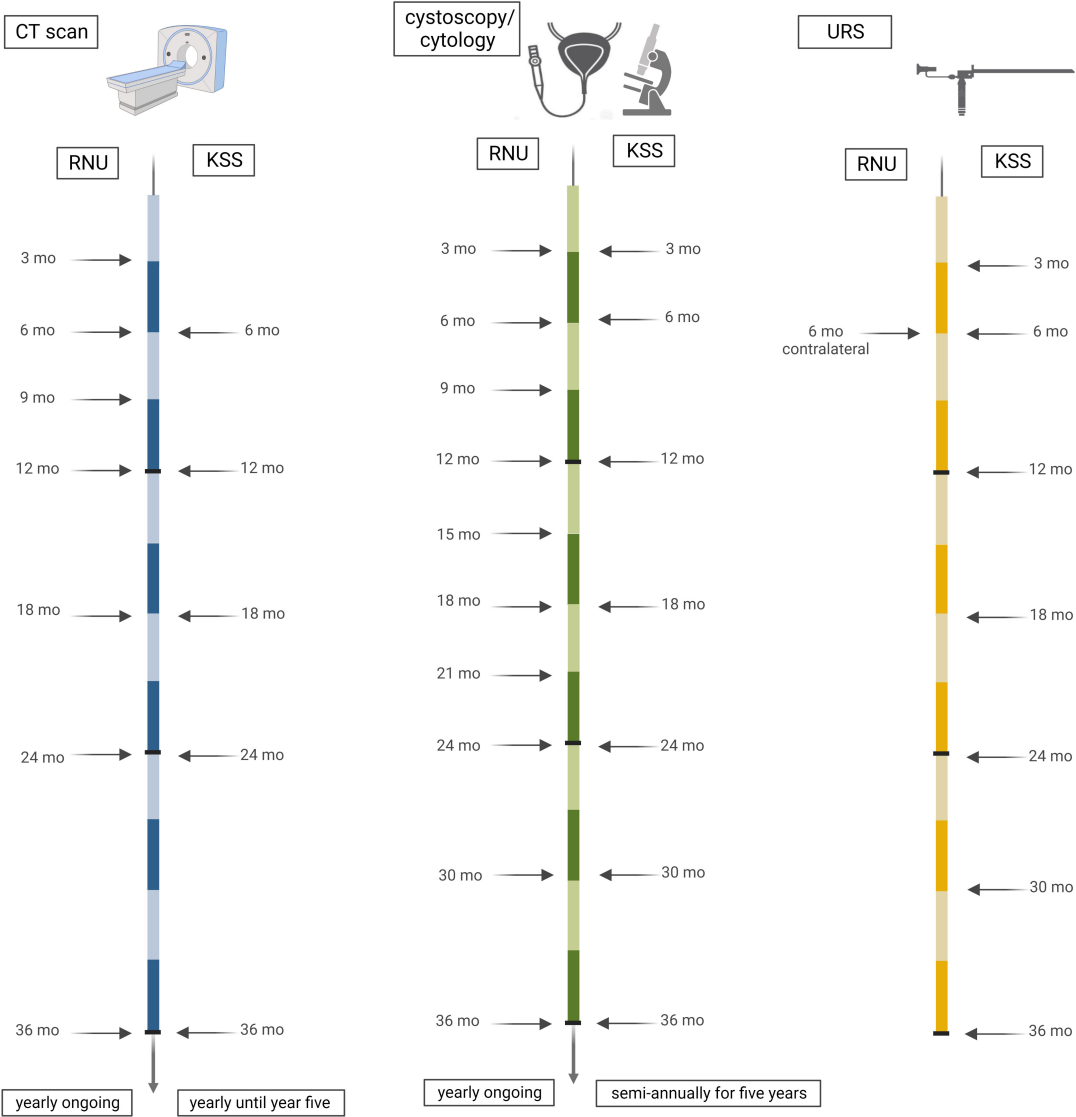
Timeline of proposed follow-up. Based on our cohort data, we suggest three-monthly cross-sectional imaging in the first year after RNU, with six-month intervals in the second year and annual CT scans thereafter. After KSS, a CT should be performed every six months for two years of FU. Cystoscopy is proposed every three months for the first two years after RNU, semi-annually in year three, and yearly ongoing. In KSS, cystoscopy should be performed at three and six months and semi-annually thereafter for five years, and ipsilateral URS should be performed at three and six months and semi-annually thereafter for three years. Contralateral URS is recommended at six months after RNU.

The rate for distant metastasis after RNU was 38.9%, with 85.7% occurring in the first year of FU, 9.5% in year two, and then only 4.8% in year three. Thus, we reinforce our support for the imaging FU regime of current EAU guidelines (1) and propose close computer tomography (CT) scans for two years after RNU with following yearly intervals. However, the contralateral UUT recurrence rate was 7.4% after RNU, occurring after a mean of 3.7 months (range 0-9), although all the cases were detected by imaging, which may suggest the need for URS at least once in the first 3 to 6 months postoperatively (**Figure 4**). After receiving KSS in low-risk disease, recurrence was apparent in 57.1% of cases, with a consistent yearly rate from 12.5% to 20.5% for up to five years after surgery. Additionally, the resection status of the patient should not be underestimated, as the R classification has considerable clinical significance as a strong predictor of prognosis related to its location and multifocality (30, 31). A recent multicentric study has investigated the oncological outcomes of positive margins after radical cystectomy for bladder cancer, with 50% of patients recurring within one year after surgery. The multifocality of positive margins presented with the worst prognosis (32). These data should be considered when suggesting UTUC FU regimens, after which there should be closer monitoring in the case of an R1 or R2 status.

Two patients in our cohort presented with sarcomatoid differentiation, and both were diagnosed with an advanced tumor stage at diagnosis, followed by death within one year after surgery. In contrast to the proposed FU protocol of the current EAU guidelines (1), our data propose an intensified and close-meshed continuation of semi-annual cystoscopies for this period of time. Metastasis occurred in year one and two after surgery; identification of these should be covered with the recommended intervals for CT imaging at six months for two years and with annual CT scans thereafter up to year five (1). Recurrence in the ipsilateral UUT occurred in up to a mean time of three years postoperatively, with a large range of recurrence time. This suggests an intensification of URS from the recommended surveillance protocol, once after three months only (1) followed by semi-annually for three years (**Figure 4**).

In line with the current EAU guidelines (1), we propose a strict imaging protocol in the first two years with following yearly intervals to exclude distant metastases. In KSS, we recommend strict semi-annual cystoscopies for up to five years, due to the continuous risk of bladder recurrence, and regular URS for three years. In RNU, we suggest a decrease of cystoscopies to yearly intervals after year three. Contralateral UUT after RNU should be considered once in the first three to six postoperative months.

In bladder cancer, variant histologies were found in up to 25% of cases, with observed worse survival in plasmacytoid, small-cell, and sarcomatoid differentiation (33). Regarding UTUC, a recent study has showed an association between variant histology and advanced stage and poor survival. However, diagnosis of UTUC with variant histologies presented with a significantly higher tumor stage. The authors therefore concluded that variant histology on surgical pathology does not surely provide additional prognostic information due to the bias of the higher found tumor stage (34). This underlines the importance of accurate pathological diagnosis and a focus on larger studies with close systematic FU, as proposed in our work, to implement a perhaps even more intensive and prolonged FU in variant histologies if needed.

Our study does have limitations. Due to the rarity of the disease, the number of patients affected is generally low and is further reduced when a sufficiently long follow-up period is required. Another limitation of the study notably includes its retrospective character and low sample size, especially in the KSS group. Finding adequate follow-up regimens on the basis of small patient cohorts remains difficult to implement. Moreover, with a low level of evidence for follow-up recommendations, there is no wide agreement on the invasiveness performed in the oncological aftercare setting.

## Conclusion

In rare and aggressive UTUC, strong evidence is required for creating accurate and individual surveillance strategies, depending on the tumor stage and surgical intervention. Our data strengthen the FU recommendations of the current EAU guidelines, including regular CT imaging for 2 years and then yearly. Cystoscopy can already be switched to yearly examinations in RNU after year three of FU. In contrast, KSS requires intensification of cystoscopies due to a high constant recurrence rate of up to over five years. The URS of the contralateral ureter should be proposed once at three to six months after RNU, and in KSS it must be continued for up to three years to sufficiently detect UUT recurrence, thus preventing the undetected spread of this aggressive disease.

## Data availability statement

The data analyzed in this study is subject to the following licenses/restrictions: Available with permission from corresponding author. Requests to access these datasets should be directed to renate.pichler@i-med.ac.at.

## Ethics statement

The studies involving human participants were reviewed and approved by 1053/2021. Written informed consent for participation was not required for this study in accordance with the national legislation and the institutional requirements.

## Author contributions

RP and AKL conceived and designed the research. TG, DB, and AKL performed the patient record reviews. SM and HU contributed their statistical analysis. RP, AKL, AL, MP, FK, and AS analyzed and interpreted the data. AKL, MP, and RP drafted the manuscript. MP and RP conducted supervision. All authors made substantial contributions to the manuscript draft, critically revised it, and approved the submitted final version.
